# Development of αβ and γδ T Cells in the Thymus and Methods of Analysis

**DOI:** 10.3390/ijms262411939

**Published:** 2025-12-11

**Authors:** Aleksey Bulygin, Elena Golikova, Sergey Sennikov

**Affiliations:** 1 Laboratory of Molecular Immunology, Federal State Budgetary Scientific Institution Research Institute of Fundamental and Clinical Immunology, Novosibirsk 630099, Russia; aleksej.bulygin95@mail.ru; 2 Federal State Autonomous Educational Institution of Higher Education I.M. Sechenov, First Moscow State Medical University of the Ministry of Health of the Russian Federation, Moscow 119435, Russia; elenagolikova329@gmail.com

**Keywords:** thymus, αβ T cells, γδ T cells, selection, T cells maturation, thymus analysis

## Abstract

The thymus, as the primary lymphoid organ for T cell development, orchestrates a complex continuum of processes encompassing precursor migration, lymphocyte lineage commitment, and antigen-guided selection to generate a self-tolerant and immunocompetent T cell repertoire. The thymus is anatomically divided into the cortex, which facilitates the positive selection of thymocytes through interactions between T cell receptors and self-peptide–MHC complexes on cortical epithelial cells, and the medulla, which mediates negative selection by medullary epithelial cells in concert with dendritic cells via the presentation of self-antigens. Key regulatory elements controlling thymocyte development include the transcription factors ThPOK/Runx3 and Sox13/PLZF, chemokine-driven migration mediated by CXCR4 and CCR7, and cytokine signaling. These components collectively exert a profound influence on the final outcome: the establishment of TCR affinity thresholds for tissue-specific antigens in mature T cells. In summary, the integration of multidimensional methodologies highlights the pivotal role of the thymus in immune tolerance, with translational implications for autoimmunity, cancer immunotherapy, and regenerative medicine, as reviewed herein.

## 1. Introduction

The thymus serves as a primary organ of the human immune system, responsible for maintaining immune response homeostasis and regulating the T-lymphocyte pool. The thymus reaches maturity in utero and subsequently undergoes age-related involution. This process involves architectural remodeling through the gradual replacement of thymic parenchyma with adipose and connective tissue, leading to a decline in the organ’s functional capacity [1]. Structurally, the thymus is composed of cortical and medullary zones, enclosed by a connective tissue capsule [2,3]. The cortex is predominantly populated by cortical thymic epithelial cells (cTECs) [4,5], which support the early maturation of thymocytes, TCR gene rearrangement, and positive selection. The medulla, in contrast, is primarily constituted by medullary thymic epithelial cells (mTECs) [6,7], which mediate negative selection and facilitate differentiation into CD4^+^ or CD8^+^ single-positive (SP) T cells based on TCR affinity [5]. Upon maturation, T cells migrate to secondary lymphoid organs to orchestrate immune responses.

In this review, we focus on human thymopoiesis, with relevant comparisons to murine models. The review encompasses the entry of progenitor cells, lineage commitment (to αβ versus γδ T cell lineages), selection processes, molecular regulators, and contemporary analytical methodologies. We highlight recent advances in the field, such as the role of TCR signaling gradients and mechanotransduction. Additionally, we address extrathymic T cell development and present applications in immunotherapy and transplantation, addressing current challenges associated with the thymus.

## 2. Starting Point of αβ and γδ Thymocyte Formation

First, it is necessary to discuss the fact that early T cell progenitors, phenotypically characterized as CD34^+^CD38^+^CD127^+^, migrate into the thymus at the corticomedullary junction, a process directed primarily by the CCR9/CCL25 and CCR7/CCL19/CCL21 chemokine axes expressed by thymic stromal cells [8,9]. A critical interspecies divergence is observed in the homing mechanism, which is vascular-dependent in humans; in mice, in comparison, it occurs independently of vascular pressure [10,11]. Concurrently, the upregulation of c-Kit and FLT3 in these early progenitors enhances their responsiveness to the corresponding stromal factors, SCF and FLT3L, thereby facilitating their migration and subsequent integration into the thymic microenvironment [12,13].

Upon migration of T cell progenitors into the thymus, cortical thymic epithelial cells (cTECs) activate the Notch signaling pathway via the Delta-like ligand 4 (DLL4) [14], inducing the key transcription factors TCF-1 (encoded by *Tcf7*) and GATA-3 to initiate T cell lineage commitment [15]. Concurrently, TCR gene rearrangement proceeds at the TRG (γ), TRD (δ), and TRB (β) loci, a process mediated by RAG1/2 and TdT enzymes during the double-negative (DN) stages (DN1 to DN3) of development [16]. The critical lineage choice between the αβ and γδ T cell fates is governed by a kinetic signaling model, where the signal’s duration and intensity dictate the developmental outcome: prolonged/weak signals favor the αβ lineage, whereas strong/rapid signals promote the γδ lineage [17,18]. This mouse model experiment is further supported by evidence demonstrating that T cell lineage specification can occur independently of the TCR type itself, being primarily determined by signal strength [19].

Following successful αβ pre-TCR assembly (composed of pTα and TRB chains) at the DN3 stage, a signaling cascade is initiated through the kinases LCK and ZAP70, triggering a robust proliferative burst and facilitating the transition from the double-negative (DN) to the double-positive (DP) stage, a process mediated by the ERK/MAPK pathway [20,21]. In DP thymocytes, recombination of the TRA (α) locus occurs, generating a complete αβTCR complex that is then poised for subsequent selection processes [22,23]. Conversely, commitment to the γδ T cell lineage is driven by a strong CD3-mediated signal, which activates the ERK/MAPK pathway. This signaling induces the expression of the key transcription factors PLZF and Sox13, which act to suppress the genetic program for αβ lineage development and prepare γδTCR-expressing thymocytes for their specific selection [24]. In contrast, the αβ lineage relies on the expression of Bcl11b, a factor that represses genes associated with the γδ fate [25]. The pre-selection events at the DN3 stage are therefore critical for determining thymocyte fate, as it is during this phase that the fundamental αβ and γδ TCR chains are assembled, setting the stage for their subsequent evaluation during the distinct selection processes within the thymus.

For the subsequent discussion in this review, the processes will be delineated based on the fundamentally divergent differentiation pathways undertaken by DP thymocytes bearing human αβ or γδ T cell receptors. The distinct developmental trajectories and selection mechanisms for αβ and γδ T cells will therefore be discussed in separate, dedicated sections.

## 3. Formation αβ TCR T Cells

**Figure 1:**
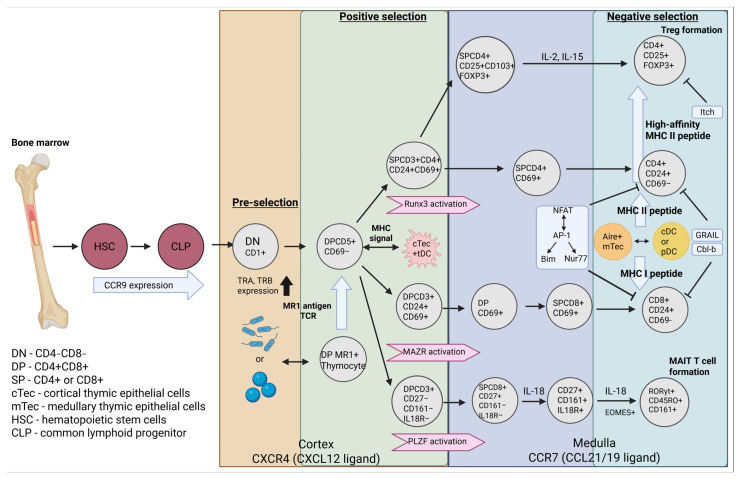
The process of differentiation of thymocytes into αβ T cells. During migration of thymocytes into the thymus, expression of TRA and TRB loci leads to the formation of the αβ TCR-CD3 complex, then the already formed thymocyte undergoes a gradual stage of positive and negative selection, during which CD4 or CD8 subpopulations of T cells are formed, possessing a function from immune response to tolerance. Tregs undergo their development through the expression of FOXP3, IL-2, and IL-15, which maintain their high TCR affinity. MAIT T cells begin their development by receiving a microbial metabolite at the DP stage, after which the formed MR1 undergoes positive selection and, mediated by the expression of PLZF and IL-18, further development.

For newly formed αβTCR thymocytes, their subsequent fate is determined by two critical checkpoints: positive and negative selection. Positive selection serves to assess the reactivity of the αβTCR to self-peptide–MHC (pMHC) complexes presented by cortical thymic epithelial cells (cTECs). Mechanistically, cTECs express unique proteasomes (e.g., the thymoproteasome for MHC-I) and proteases (e.g., cathepsin L for MHC-II) to generate a specialized repertoire of self-pMHC ligands [26,27]. This unique antigen-processing machinery enables the thymus to evaluate the signal strength transmitted by the engaged TCR (Figure 1). For instance, interactions of intermediate affinity governed by TCR-pMHC recruitment frequency and digital sensitivity gradients rescue thymocytes from death by neglect [28]. Notably, studies of TCR mechanotransduction reveal that high-affinity interactions form stable bonds under physiological forces, sustaining lifetimes of several seconds even with micromolar equilibrium dissociation constants (*K_D_*) [29].

Following positive selection, the lineage commitment of thymocytes is determined by the strength and context of TCR-pMHC interactions, which establish a reciprocal regulatory axis between the transcription factors ThPOK and Runx3. A weak but sustained TCR signal, typically resulting from MHC-II engagement, activates the LCK/ZAP70/calcineurin/NFAT signaling cascade, inducing the expression of ThPOK (encoded by *Zbtb7b*), which directs CD4^+^ lineage commitment by directly repressing Runx3 transcription and simultaneously activating CD4^+^ lineage genes, such as Cd4 and Foxp3, via recruitment of histone acetyltransferases (HATs) [30,31]. Conversely, TCR engagement with MHC-I, often involving stronger signals and β-catenin accumulation, promotes Runx3 upregulation. Runx3 then orchestrates CD8+ lineage commitment by binding to enhancers of the Cd8a and Cd8b1 genes to activate their transcription, while simultaneously repressing *Zbtb7b* through the recruitment of histone deacetylases (HDACs). Furthermore, Runx3 induces the expression of Eomesodermin (Eomes), a critical factor for the development of cytotoxic function in CD8^+^ T cells [32,33]. Thus, the post-positive selection fate of a single-positive (SP) thymocyte is ultimately determined by the competitive balance between ThPOK and Runx3, which reinforces the chosen lineage and suppresses the alternative pathway [34,35].

A key observation during the maturation of single-positive (SP) thymocytes is the concurrent upregulation of surface proteins CD24 and CD69, which serve as markers of successful proliferation and readiness for migration [36,37]. In addition to these phenotypic changes, SP thymocytes undergo a chemokine receptor switch that facilitates their relocation: they downregulate CXCR4, which responds to CXCL12 and retains thymocytes in the cortex, while concurrently upregulating CCR7. This enhanced expression of CCR7 enables their chemotactic entry into the medulla, a process mediated by ligands CCL19 and CCL21 expressed by medullary thymic epithelial cells (mTECs) (Figure 1) [38,39].

The process of negative selection is implemented within the thymic medulla, typically occurring during the transition of thymocytes from the DP to the semi-mature CD24+ SP stage [40]. This selection is critically regulated by the interplay between medullary thymic epithelial cells and dendritic cells (DCs). mTECs, through the expression of the transcriptional regulator Aire (Autoimmune Regulator), promote the expression of a broad repertoire of tissue-specific antigens (e.g., insulin and thyroglobulin) via chromatin remodeling, thereby presenting these self-antigens primarily in the context of MHC-II [41]. Conversely, DCs contribute by cross-presenting antigens derived from degraded mTEC proteins on MHC-I molecules [42] (Figure 1). The role of Aire^+^ mTECs is pivotal in purging the emerging T cell repertoire of autoimmune potential [41,43], with DCs also assuming a broad role in the deletion of autoreactive thymocytes (Figure 1) [44]. Autoreactive thymocytes receiving a strong TCR signal initiate the calcineurin-dependent NFAT pathway. In conjunction with AP-1 (*Fos*/*Jun*) [45], this induces the expression of pro-apoptotic proteins such as Bim (*Bcl2l11*) [46] and Nur77 (*Nr4a1*) [47]. Bim triggers the mitochondrial apoptotic pathway by compromising membrane integrity, leading to cytochrome c release and subsequent activation of caspases-9 and -3 [48]. Alternatively, in the absence of CD28 co-stimulation (e.g., due to low B7 expression on antigen-presenting cells), TCR signaling can induce clonal anergy. This state of functional inactivation is mediated by E3 ubiquitin ligases like Cbl-b and GRAIL, which degrade key signaling molecules such as CD3ζ and PKCθ [49,50] (Figure 1). For instance, the work of Wang and colleagues demonstrated that inhibiting Bim expression impaired the deletion of autoreactive thymocytes, leading to their escape from apoptosis [51]. A small fraction of autoreactive T cells may evade clonal deletion and enter a state of anergy, characterized by suppressed IL-2 production and heightened expression of inhibitory receptors such as PD-1 and CTLA-4. However, under certain conditions such as inflammation, these cells can be reactivated by cytokine-driven proliferation (e.g., via IL-7 and IL-15) and may subsequently undergo peripheral deletion in secondary lymphoid organs where DCs present tissue-specific antigens.

A key and unique outcome of negative selection is the diversion of a subset of high-affinity, autoreactive T cells from clonal deletion towards a specialized fate as regulatory T cells (Tregs). These cells are generated precisely as a consequence of high-affinity TCR interactions with self-antigens presented during the negative selection process. In contrast to conventional CD4^+^ T cells, these precursors escape apoptosis through a combination of intense TCR and CD28 co-stimulatory signaling, which activates the transcription factor FOXP3. FOXP3 serves as the master regulator and definitive molecular marker for Treg lineage commitment and function [52,53] (Figure 1). It is noteworthy that a population of Tregs with intermediate TCR affinity also exists and plays a critical role in maintaining immune homeostasis by suppressing other autoreactive T cells, thereby providing a complementary mechanism for peripheral tolerance [54]. A key event in Treg stabilization is the upregulation of CD25 (IL-2Rα), the high-affinity subunit of the IL-2 receptor. This process enables Tregs to compete for IL-2, leading to the activation of the transcription factor STAT5, which in turn reinforces and stabilizes FOXP3 expression, cementing the Treg differentiation program [55] (Figure 1). Ultimately, the high-level expression of FOXP3 drives the functional specialization of Tregs into potent inducers of immune tolerance, whose primary role is the suppression of immune responses against self-antigens.

Another distinct population of αβ T cells that undergoes a multi-stage thymic selection process is the Mucosal-Associated Invariant T (MAIT) cell lineage. MAIT cells constitute a distinctive T cell subset that bridges innate and adaptive immunity, primarily specialized in mounting responses against bacterial pathogens [56]. Their development is initiated by a specific TCR gene rearrangement, leading to the expression of an invariant TCR α-chain (Vα7.2-Jα33) paired with a limited repertoire of TCR β-chains [57,58]. Subsequently, MAIT cell precursors undergo a unique thymic selection process mediated by the MHC class I-related protein 1 (MR1). MR1 ligands are broadly classified into endogenous metabolites and microbial products; their origin dictates compartmentalized function. During this selection process, TCR affinity for MR1-presented mucosal or endogenous metabolites is evaluated rather than the canonical self-pMHC complexes that select conventional αβ T cells [59] (Figure 1). A remarkable feature of this process is the ability of MR1 to present a broad spectrum of antigenic ligands, ranging from riboflavin (vitamin B2) biosynthesis intermediates to other vitamin B-derived metabolites [60]. Furthermore, environmental factors can modulate this system; for instance, recent research in mice has demonstrated that tobacco smoke, which contains numerous xenobiotic compounds, can upregulate MR1 surface expression and alter MAIT cell effector function [61]. The transcriptional programming and functional maturation of MAIT cells are regulated by factors such as the transcription factor PLZF and potentially miR-181a/b-1, which promote an innate-like effector phenotype and migratory readiness [62,63] (Figure 1). Following positive selection via MR1, MAIT cells complete their final maturation, transitioning from a CD24^+^CD44^−^CD27^−^CD161^−^ to a CD24^−^CD44^+^CD27^+^CD161^+^ phenotype as they migrate to peripheral tissues in response to signals such as IL-18 and microbial stimulation [59] (Figure 1). In summary, the developmental pathways of αβ T cells exhibit remarkable plasticity, encompassing a wide spectrum of mechanistic adaptations—as exemplified by MAIT cells—to tailor immune responses to specific antigenic challenges.

In conclusion, negative selection emerges as a multi-layered process that integrates TCR signaling, co-stimulatory inputs, and complex transcriptional networks to eliminate autoreactive T cell clones. The flexibility of its outcomes—ranging from clonal deletion and anergy to the diversion of cells into regulatory T cell (Treg) and Mucosal-Associated Invariant T (MAIT) cell lineages—collectively safeguards the critical balance between self-tolerance and effective immune defense.

## 4. Formation γδ TCR T Cells

**Figure 2:**
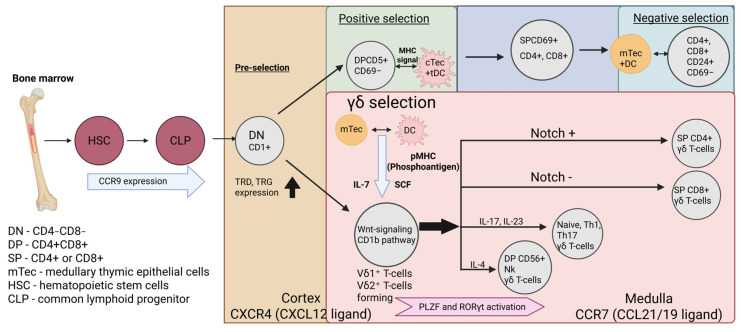
The process of thymocyte proliferation into γδ T cells. Unlike αβ T cells, γδ T cells undergo selection with increased expression of the TRD and TRG loci, which determine the fate of T cells along the γδ lineage. During selection in the triad between mTEC, tDCs and thymocytes, antigen presentation and signal induction from TCR via TFC-1 Wnt signaling via the CD1d-dependent pathway occur, during which, upon induction of transcription factors, γδ T cells differentiate into various subpopulations.

In contrast to the αβ T cell developmental pathway, which is defined by discrete positive and negative selection checkpoints, γδ T cells diverge early at the DN1a/b to DN3 stages, undergoing a more permissive and subset-dependent selection process. Their commitment is driven by simultaneous TRG and TRD gene rearrangement, which generates strong CD3-ERK/MAPK signals that instruct lineage specification [64,65]. A fundamental distinction from αβ TCRs lies in their antigen recognition strategy. Unlike the strict peptide–MHC restriction of αβ T cells, the γδ TCR, formed by an alternative architecture of γ- and δ-chains and influenced by early Sox13 expression, confers the ability to perceive a broader spectrum of antigens in an MHC-independent manner. This remarkable property enables the recognition of both exogenous and endogenous ligands, including foreign and self-antigens such as phosphoantigens [66]. The selection process for γδ T cells occurs in distinct phases (Figure 2). Initially, CCR9-expressing γδ thymocyte precursors interact with mTECs that secrete IL-7 [67] and SCF [68] into the microenvironment, promoting survival and proliferation. Subsequently, mesenchymal stromal cells provide Wnt ligands to sustain the γδ lineage through β-catenin and transcription factor TCF-1 activation [69]. A critical event at this stage is the regulation of two key transcriptional determinants, PLZF (Promyelocytic Leukemia Zinc Finger) and RORγt (Figure 2). The balance of these factors dictates the ultimate fate and phenotype of mature γδ T cells, polarizing them towards distinct subsets such as Vδ1^+^ (TRDV1^+^) or Vδ2^+^ (TRDV2^+^) cells. The activity of PLZF and RORγt also upregulates the production of IL-17 and IL-23, key cytokines that equip the γδ T cell population with its immune potential prior to peripheral migration [24,70,71]. It has been demonstrated that the outcome of γδ T cell differentiation in the thymus, regulated by the Notch signaling pathway, is directly dependent on the strength of the signal originating from TCR. A gradient of TCR signal strength dictates the choice between alternative differentiation lineages: weak signals lead to the formation of IL-4-producing Vγ1^+^ γδ T cells, whereas strong signals induce the development of IL-17-producing Vγ2^+^ γδ T cells [72]. Thus, Notch-mediated regulation, contingent upon TCR signal strength, drives the generation of both NK-like and helper-like γδ T cells. Furthermore, contrary to the prevailing view that γδ T cells are double-negative for the CD4 and CD8 co-receptors, evidence indicates that upon antigenic stimulation via the TCR in combination with Notch signaling, γδ T cells can upregulate the expression of either the CD4 [73] or CD8 [74] co-receptors, thereby demonstrating the plasticity of the CD4^+^ or CD8^+^ γδ TCR T cell phenotype (Figure 2). Thus, γδ T cell selection is characterized by permissive TCR interactions that guide subset-specific functional programming, rather than enforcing stringent self-tolerance checkpoints.

γδ T cell selection in the thymus is now understood to be permissive rather than strictly dichotomous. In comparison to αβ T cells, the thymic selection of γδ T cells can be viewed as affinity-based screening, whereby high-affinity clones develop into distinct T cell populations, whereas low-affinity clones are eliminated through apoptosis or can be converted to Vδ2^+^ T cells with antigen-presenting capabilities. Lastly, the functional maturation of selected γδ T cells involves interaction with a combined response from mTECs and dendritic cells. This engagement, often mediated through BTNL proteins, triggers ERK/MAPK signaling, refining the repertoire towards functional effector clones [75].

### 4.1. Vδ1^+^ T Cell Population

Vδ1^+^ T cells are generated during the early DN1-DN2 thymocyte stages and express a TCR incorporating the Vδ1 chain. Their development is influenced by IL-7 and Wnt signaling, which activate the transcription factor TCF-1 via a CD1d-dependent pathway [76]. Following their thymic development, Vδ1^+^ T cells migrate to peripheral tissues, where they reside and function as sentinels at barrier sites such as the skin, mucosa, and intestine [77,78]. Their homing to these locations is directed by specific chemokine axes; for instance, keratinocyte-derived CCL20 (MIP-3α) and CCL27 (CTACK) activate CCR6 on Vδ1^+^ T cells to guide their migration [79]. Similarly, CXCR16 expression by mucosal cells facilitates the recruitment of CXCR6-expressing Vδ1^+^ T cells [80]. Functionally, this subset is primarily responsible for reinforcing immune barriers at epithelial surfaces. They achieve this through the production of IL-17 and IL-22, cytokines that are crucial for maintaining mucosal integrity and defense against pathogens [81]. Concurrently, Vδ1^+^ T cells also exhibit potent cytotoxic activity. Via the expression of receptors such as NKG2D and DNAM-1, they are capable of eliminating infected and malignant cells [82,83].

This unique combination of barrier surveillance and effector functions currently makes Vδ1^+^ T cells a subject of intense scientific interest, highlighting their significant potential for application in personalized immunotherapeutic strategies.

### 4.2. Vδ2^+^ T Cell Population

The Vδ2^+^ T cell subpopulation develops later than its Vδ1^+^ counterparts, primarily originating from DP thymocytes that express a TCRγ9δ2 receptor. Their development is driven by phosphoantigens presented by DCs and mTECs [84,85]. A notable feature of these cells is their capacity to function as APCs even prior to peripheral migration. Selected, thymus-matured Vδ2^+^ T cells can leverage their unique TCRγ9δ2 to acquire and present antigens, thereby acting as non-conventional APCs [86,87]. Following their thymic egress, Vδ2^+^ T cells predominantly circulate in the blood and lymphoid organs. Their homing to sites of inflammation is guided by the expression of chemokine receptors such as CCR5 (binding RANTES) and CXCR3 (binding CXCL11) [88,89]. Due to their antigen-presenting capability, Vδ2^+^ T cells often play an immunostimulatory role by activating αβ T cells through antigen cross-presentation via MHC-I molecules [90]. Furthermore, Vδ2^+^ T cells are potent effectors in antimicrobial and antitumor immunity. Upon recruitment to inflammatory foci, they secrete key pro-inflammatory cytokines, including IFN-γ and TNF-α, and directly mediate the lysis of target cells through the release of cytolytic molecules such as perforin and granzymes [91].

Thus, the unique developmental pathway of γδ T cells endows them with a broad and versatile immunological repertoire, encompassing hybrid innate–adaptive functions, diverse antigen recognition and presentation capabilities, and the capacity to recruit and modulate αβ T cell responses, collectively positioning them as pivotal regulators of integrated immune surveillance.

## 5. Affinity and Its Role in T Cell Formation

The interaction strength between the T cell receptor and the peptide–MHC complex is a primary determinant of T cell efficacy in antigen recognition and response. TCR affinity is a decisive factor in determining the fate of individual T cell clones and in maintaining overall immune homeostasis. Consequently, significant research efforts are now focused on elucidating the precise mechanisms by which the thymus and its cellular constituents interpret this affinity threshold to shape the entire peripheral T cell repertoire.

The binding affinity of T cell receptors is conventionally quantified using dissociation and association rates (k_off_ and k_on_, respectively) and the equilibrium dissociation constant (*K_D_*). These parameters define the strength, duration, and kinetics of the TCR-pMHC interaction [92,93]. At the molecular level, TCR-pMHC binding is often assessed using solution-based surface plasmon resonance (SPR), which measures 3D binding affinity. A significant limitation of this approach, however, is its inability to account for the effects of TCR-pMHC rebinding kinetics (k_on_) under physiological, two-dimensional (2D) conditions on the cell membrane [94]. To address this limitation, novel tetramer-based technologies, such as NTAmer assays, have been developed to evaluate both the strength and repetitiveness of TCR-pMHC engagements in a more biologically relevant context [95]. For γδ T cells, affinity is typically determined using tetramers loaded with phosphoantigens, with binding strength quantified by means of atomic force microscopy [96]. The application of these diverse methodologies for investigating intermolecular interactions across human T cell populations of varying affinity has been instrumental in identifying key biophysical and molecular factors that govern antigen recognition (Table 1).

To complete the signaling landscape characterized by each affinity threshold, it is established that a high-affinity TCR interaction is defined by stable binding to pMHC complexes, a feature often associated with autoreactive potential. Such engagements trigger intense signaling cascades: Lck/ZAP70-mediated phosphorylation of CD3ζ ITAMs initiates calcineurin-dependent activation of NFAT and AP-1 (*Fos*/*Jun*). These factors subsequently induce the expression of pro-apoptotic genes, including Bim and Nur77, through their specific enhancer elements, ultimately leading to clonal deletion in the thymic medulla [97]. Concurrent activation of the ERK/MAPK pathway further reinforces negative selection by upregulating anergy-associated markers such as PD-1 and CTLA-4 [98].

In the case of γδ T cells, selection is modulated by interactions with BTNL proteins on mTECs, which facilitate γδTCR engagement and signal transduction [99]. Furthermore, the transcription factor TOX (Thymocyte Selection-Associated HMG Box) plays a critical role in mediating the apoptotic fate of self-reactive thymocytes by regulating the expression of Bim [100]. Consequently, only the high-affinity γδ T cell clones, such as the Vδ1^+^ subset, are positively selected to mature and fulfill their pivotal role in adaptive immunity.

Notably, an intermediate TCR affinity is optimal for generating a functional T cell repertoire. TCR signaling, when coupled with CD28 co-stimulation, activates the PI3K-AKT-mTOR pathway, which drives proliferation and differentiation into effector subsets such as Th1, Th17, and cytotoxic CD8^+^ T cells. This process involves the induction of lineage-defining transcription factors: T-bet (for Th1) via STAT4 and RORγt (for Th17) via STAT3. Simultaneously, TCF-1 (encoded by *Tcf7*) and LEF-1 support clonal expansion by modulating chromatin accessibility at key cytokine loci (e.g., IFN-γ and IL-17) [101,102]. T cells with intermediate affinity sustain CD25 (IL-2Rα) expression, thereby enhancing their survival through IL-2 signaling. Furthermore, the sustained expression of CD69, induced via the NFAT pathway, serves as a key marker of successful thymic selection and activation [103]. The critical balance of CD28 and CD69 signaling is essential; disruptions in this equilibrium can cause a failure in positive selection, leading to apoptosis and resulting in immunodeficiency [104,105].

Finally, weak TCR signaling, characteristic of low-affinity TCR interactions, is a hallmark of naive T-lymphocytes and specific regulatory subsets. These low-intensity signals activate Foxo1, which inhibits the mTOR pathway and induces the expression of Klf2. This transcription factor upregulates the homing receptors CCR7 and S1PR1, facilitating the egress and circulation of naive T cells. In the context of low-affinity regulatory T cells (Tregs), the master regulator Foxp3 suppresses pro-inflammatory genes (e.g., *IL-2* and IFN-γ) and activates suppressive molecules (e.g., *CTLA-4* and *IL-10*) by recruiting chromatin-modifying enzymes such as HDACs and EZH2 to target gene promoters [54,106,107]. Low-affinity signaling is also intrinsically linked to the induction of anergy. TCR engagement in the absence of co-stimulation activates E3 ubiquitin ligases such as Cbl-b and GRAIL, which degrade key signaling molecules such as CD3ζ and PKCθ, thereby rendering the cell unresponsive [108,109]. The transcription factor NR4A serves as a marker for cells that have escaped deletion but remain in a state of tolerance.

Among γδ T cells, a low-affinity developmental path is associated with the expression of PLZF (*Zbtb16*), which dictates a tissue-resident phenotype, as seen in many Vδ2^+^ T cells. These low-affinity γδ T cells can subsequently participate in local immune responses by presenting phosphoantigens in the periphery [110,111].

In summary, the TCR binding affinity serves as the principal determinant orchestrating T cell fate during thymic development. It directly governs the survival and maturation of thymocytes, dictates the outcomes of negative selection, and directs the lineage choice between clonal deletion, anergy, and regulatory T cell differentiation. Collectively, these affinity-driven processes are fundamental in shaping a functional and self-tolerant T cell repertoire.

## 6. Methods for Analyzing the Processes of T Cell Formation in the Thymus

The seminal interest in thymic function emerged in the early 1960s, when Jacques Miller, through thymectomy experiments in mice, discovered its pivotal role in immunity and identified T-lymphocytes [112]. By the 1990s and early 2000s, researchers had delineated the thymic architecture and stromal components critical for T cell selection, alongside key regulatory proteins such as Aire, which governs the presentation of self-antigens by thymic dendritic cells and the pMHC-TCR axis.

The advent of advanced TCR affinity assessment tools—including tetramer technology and microfluidic platforms—has aided in elucidating the mechanisms underlying the selection of T cells based on high or low TCR affinity, in addition to the deletion of autoreactive clones. Furthermore, the rise of sequencing technologies, particularly bulk and single-cell RNA-seq, has enabled comprehensive analysis of cellular transcriptomes. These methods have been instrumental in revealing regulatory pathways and delineating the heterogeneity of thymic cell populations at single-cell resolution. For instance, scRNA-seq has refined the classification of γδ T cell subsets, such as Vδ1^+^ and Vδ2^+^ lineages [113]. Thus, technological progress has provided a comprehensive toolkit for dissecting thymic processes, greatly advancing our understanding of its role in both health and disease.

### 6.1. Experimental Models for Studying Thymopoiesis In Vitro

**Figure 3:**
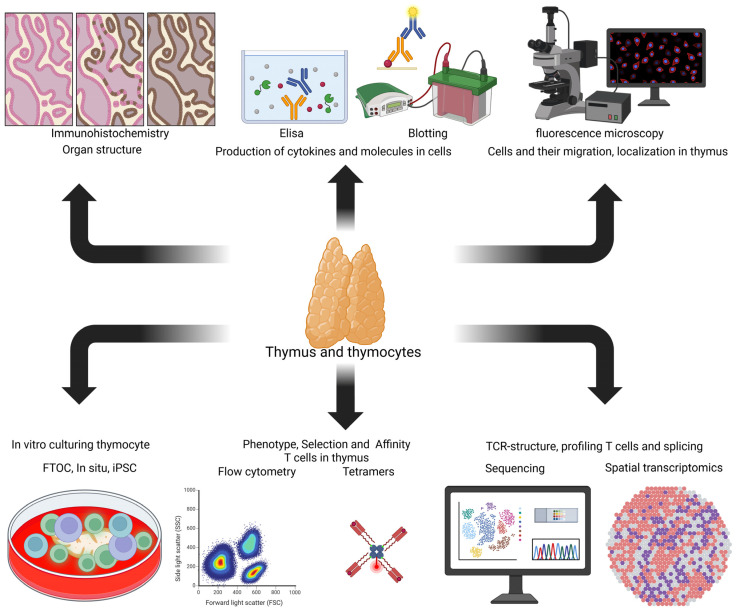
Methodologies employed for the analysis of human and mouse thymic tissue. Experimental models of thymopoiesis in vitro help to easily reproduce situations occurring in the thymus (formation, selection, etc.). Tetramers and flow cytometry help to determine the stages of thymus cell differentiation and determine their functionality. The use of sequencing and multiomics provides a complete picture of the architecture of formed TCR αβ and γδ T cells and, simultaneously, determines alternative splicing in differentiating T cells. IHC and fluorescence microscopy provide opportunities to study the dynamics of thymocyte distribution and their interaction with thymic cells. Immunoblotting and ELISA aid in studying the production of cytokines and various isoforms of key transcription factors that are crucial for thymocyte differentiation at all stages of development in the thymus.

The study of T cell selection processes in the thymus actively involves complex experimental approaches that enable the reproduction of key differentiation stages in vitro (Figure 3). The main methods employed are as follows:

Fetal Thymus Organ Culture (FTOC): The application of this technique, which maintains the natural 3D architecture of both human fetal and neonatal mouse thymus, has been pivotal in elucidating the indispensable function of the stromal microenvironment. The results of studies involving FTOC have demonstrated that stromal-derived cues and cytokines, such as IL-7, are essential for supporting thymocyte proliferation and survival during the early DN-to-DP transition [114].

Reaggregate Thymus Organ Culture (RTOC): In contrast to FTOC, the RTOC methodology involves the initial disaggregation of thymic tissue followed by the re-aggregation of its cellular components. This key feature provides a unique platform for manipulating the cellular composition (e.g., by adding or removing specific stromal cell types) and genetic background (e.g., by introducing mutations). The capacity for such precise manipulation in RTOC has enabled direct experimental proof of the functional role of specific molecules, such as the Aire protein in mTECs, in mediating negative selection and establishing central immune tolerance [115].

Thymus Slice Culture (in situ model): This contemporary approach utilizes 200–400 μm thick sections of an intact thymus, prepared using a vibratome. The slices are cultured on porous membranes in a specialized medium, often supplemented with IL-7 and Stem Cell Factor (SCF). Its primary advantage lies in the preservation of the organ’s intricate 3D architecture and native stromal interactions, which are unattainable in fully dissociated systems. Fluorescently labeled progenitor cells (e.g., DN cells: CD4^−^CD8^−^CD44^+^CD25^+^) can be introduced into these slices, enabling real-time tracking of their differentiation and migration. As the model that best preserves the native architecture and cellular crosstalk, it has provided key evidence for the spatiotemporal organization of differentiation processes, including elucidating the role of the CCL21/CCR7 gradient in guiding thymocyte migration and revealing a two-step mechanism for Treg cell development that depends on integrated TCR and IL-2/STAT5 signaling within their natural niche [116,117,118].

Artificial Thymic Organoids (ATOs) and iPSC-derived Models: Recent advances in the generation of artificial thymic organoids (ATOs) and models derived from induced pluripotent stem cells (iPSCs) have provided unique platforms for modeling human thymopoiesis in vitro. These self-organizing three-dimensional structures mimic the native thymic microenvironment, effectively supporting the full spectrum of T cell development, including the generation of conventional αβ T cells, various γδ T cell subsets, and the evaluation of T cell maturation, thereby enabling the identification of key factors governing T cell differentiation [119,120,121]. These patient-specific models offer powerful tools for investigating T cell immunity, modeling genetic defects, and advancing the development of novel T cell-based therapies.

### 6.2. Analytical Methods for Studying the Thymus

A deep study of the various stages of differentiation and functions of thymus cells relies on a wide range of analytical techniques (Figure 3):

Flow Cytometry and Fluorescence-Activated Cell Sorting (FACS): Established flow cytometry protocols serve as a fundamental tool for identifying and isolating thymocyte subsets at distinct developmental stages. For instance, the use of biotinylated antibodies against CD69 and CD5 enables the efficient isolation of T cell fractions that have undergone negative selection. Multidimensional analysis combining markers such as CD24, CD4, CD8, and CD5 enables precise resolution of cell populations with low, intermediate, and high expression levels of key surface molecules, thereby facilitating the characterization of subtle differentiation transitions [122].

T Cell Receptor Sequencing (TCR-Seq): This technique enables comprehensive analysis of the clonality and diversity of the T cell repertoire. For example, the application of TCR-Seq to thymic cells has proven instrumental in characterizing V(D)J recombination events, tracking clonal dynamics during positive and negative selection, and identifying potentially autoreactive T cell clones [123]. In addition, an in situ TCR-seq approach aided in delineating significant variations in the T cell receptor (TCR) repertoire throughout T cell maturation, effectively mapping onto the canonical differentiation routes towards CD4^+^ and CD8^+^ fates [118]. The advent of TCR sequencing has further unlocked the potential to trace the evolving landscape of immune responses, thereby cementing its utility as a critical tool for personalized therapeutic interventions.

Single-Cell RNA Sequencing (scRNA-seq): This revolutionary technique has unveiled previously unrecognized heterogeneous populations and transcriptional trajectories during thymic development. For instance, scRNA-seq has refined the classification of γδ T cell subsets (e.g., Vδ1^+^ vs. Vδ2^+^) and revealed the presence and functional contributions of non-thymic lineages, such as dendritic cells and monocytes, within the thymic microenvironment [124]. Furthermore, the method is applied to investigate alternative splicing events during T cell differentiation, providing deeper insights into post-transcriptional regulation [125,126]. By capturing and analyzing full-length transcriptomes, the technique is increasingly applied to profile alternative splicing dynamics and isoform usage throughout T cell differentiation. This capability provides a deeper, more nuanced insight into the complex layer of post-transcriptional regulation that governs cell fate decisions, complementing the data on gene expression levels with information on the diversity of protein-coding messages generated [125,126]. The integration of these multifaceted data solidifies scRNA-seq as a cornerstone technique for constructing a comprehensive and high-resolution map of thymopoiesis.

Single-Cell Multi-Omics: Contemporary approaches integrate transcriptomics, cell surface protein analysis (CITE-seq), and epigenomics (scATAC-seq) within a unified analytical framework. These methods provide a comprehensive view of the interconnected regulatory networks governing cell fate decisions. For instance, the combined application of scRNA-seq and scATAC-seq has elucidated the epigenetic mechanisms—specifically, dynamic changes in chromatin accessibility—that underlie the lineage commitment to either the CD4^+^ or CD8^+^ T cell fates, revealing how key transcription factors such as ThPOK and Runx3 are regulated [127,128]. Ultimately, the systematic integration of these complementary data modalities is transforming our understanding of thymopoiesis by moving beyond correlative observations to construct causal models of gene regulatory network operation and cell identity establishment.

Spatial Transcriptomics/Multi-Omics: This approach represents a critically important contemporary technique that preserves the spatial context of molecular data. Spatial transcriptomics enables the direct correlation of gene expression patterns with a cell’s location within specific thymic niches (e.g., cortex and medulla), which is essential for deciphering the molecular foundations of cellular migration, selection, and intercellular communication. The application of this technique has been pivotal, for instance, in defining a specific niche within the thymic medulla that supports the formation of germinal centers and promotes the development of autoimmune responses in myasthenia gravis-associated thymoma [129]. Consequently, by integrating molecular profiling with histological structure, spatial transcriptomics transcends the limitations of dissociative techniques, offering a systems-level framework to resolve the spatial orchestration of immunity and its dysregulation in disease.

Immunohistochemistry (IHC) and Fluorescence Microscopy: These methods are indispensable for analyzing the spatiotemporal dynamics of thymopoiesis. Immunohistochemistry (IHC) is a method whereby protein localization in thymic tissue is visualized using enzyme-conjugated antibodies. Formalin-fixed, paraffin-embedded sections are stained with antibodies against markers such as keratins (K5/8), immune cell antigens (CD3 and CD25), or transcription factors (Aire and Foxp3), followed by chromogenic detection. IHC has been pivotal, for example, in defining KIT expression patterns in thymic epithelial cells during carcinoma [130,131,132,133]. Complementarily, fluorescence microscopy provides high-resolution imaging of cellular structures and interactions within the intact thymic architecture. It offers critical insights into thymocyte development and the thymic microenvironment, with key applications including the characterization of mTECs and their role in T-regulatory cell development [134,135,136,137].

MHC Tetramers: The use of MHC tetramers loaded with peptide ligands of defined affinity enables the direct study of TCR engagement with its cognate pMHC complex [138,139]. For instance, stimulating thymocytes with MHC tetramers loaded with low-affinity ligands—mimicking the conditions of positive selection—demonstrated that low-affinity tetramers induce sustained, yet low-amplitude, downstream TCR signaling events, particularly through the PI3K-AKT-mTOR pathway, thereby promoting survival and lineage commitment without triggering negative selection [140].

Immunoblotting (Western blot) and Enzyme-Linked Immunosorbent Assay (ELISA): These techniques collectively enable the quantitative analysis of protein expression and cytokine secretion. Immunoblotting provides quantitative data on key signaling molecules (e.g., p-AKT and Bcl-2), transcription factors (e.g., Foxp3 and Aire), and receptors, in addition to essential post-translational modifications such as phosphorylation [141,142]. ELISA has been crucial for demonstrating specific physiological changes, such as the reduction in IL-7 production linked to the decline in double-negative thymocytes during age-related thymic involution [143]. The integrated application of these methods is a powerful strategy. For example, it has been pivotal in correlating diminished IL-7 levels with impaired downstream signaling, such as reduced STAT5 phosphorylation, across various pathological contexts [144,145,146].

## 7. Concluding Remarks and Future Perspectives

The contemporary understanding of thymus biology, achieved through an integration of sophisticated culture systems and high-resolution analytical technologies, is paving the way for novel translational applications. A primary and promising frontier is the harnessing of fundamental knowledge on thymopoiesis for the controlled de novo generation of T cells with predefined antigen specificity. This approach holds significant translational potential, particularly in two key areas: enhancing the efficacy of cancer immunotherapy through the production of T cells with high-affinity TCRs for tumor antigens and preventing transplant rejection by generating T cells tolerant to alloantigens. However, the realization of this potential faces substantial hurdles. Technical challenges include the difficulty of recreating a fully functional thymic niche in vitro—the issue of “thymic mimicry”—and limitations imposed by MHC polymorphism. Immunological obstacles involve the precise control of TCR affinity and ensuring the long-term functional stability of in vitro-derived regulatory T cells. Critical unresolved questions remain, including the precise mechanisms governing αβ/γδ lineage commitment. Ultimately, overcoming these barriers—thymic mimicry, MHC restrictions, and Treg instability—will depend on the continued advancement of techniques that can more accurately reconstruct the thymic microenvironment and provide a comprehensive analysis of the molecular processes governing thymocyte selection and differentiation. Future methodological progress is anticipated to resolve these challenges, thereby yielding powerful new therapeutic strategies for oncology and transplantation medicine.

## Figures and Tables

**Table 1:** Key points in affinity grading of αβ and γδ T cell subsets using the dissociation constant (*K_D_*). At *K_D_* < 1 μM, high-affinity αβ T cells may undergo clonal deletion. Only a small percentage of T cells, under the influence of FOXP3 locus expression, become Tregs. γδ T cells form a population of Vδ1+ T cells, which are mainly responsible for adaptive immunity and form an immune barrier in areas in direct contact with foreign antigens. At *K_D_* ~1–10 μM, in relation to naive T cells, CD4^+^ and CD8^+^ helper and effector T cells are formed, respectively. *K_D_* > 10 μM, αβ naïve T cells, and those T cells that have failed negative selection are located, which leads to anergy and, notably, in a borderline state with anergy, T cells can differentiate into Tregs. Low-affinity γδ T cells form Vδ2^+^ T cells, the function of which is mainly antigen presentation.

Affinity Level	*K_D_* Range	Outcomes/Markers		Signaling/Mechanisms		Examples/Type of Cells	
High (Strong Signal)	*K_D_* < 1 μM	αβ T cells	Clonal deletion/apoptosis (Bim/Nur77);	αβ T cells	Lack costimulation → anergy (Cbl-b/GRAIL)	αβ T cells	Autoreactive αβ T cells
			Treg induction (FOXP3/CD25)		Prolonged ERK/NFAT/AP-1		high-affinity Tregs
		γδ T cells	γδ selection (PLZF/Sox13, Bim)	γδ T cells	Modulation by BTNL and activation TOX	γδ T cells	Vδ1^+^ T cells phosphoantigen response
Intermediate (Survival)	1–10 μM	αβ T cells	Positive selection → SP (CD69+)	αβ T cells	LCK/ZAP70 → ThPOK/Runx3 balance	αβ T cells	Naive CD4^+^/CD8^+^
			migration (CCR7↑)		metabolic shift (mTOR)		low Treg suppression
		γδ T cells	γδ selection (PLZF/Sox13, Bim)	γδ T cells	Activation RORγt and Notch pathway	γδ T cells	NK, Th, Th17 and Vδ2^+^
Low (Weak Signal, anergy)	>10 μM	αβ T cells	Anergy after Negative selection (NR4A/Nur77)	αβ T cells	HDAC/EZH2 suppression	αβ T cells	Naive/low-affinity Aβ; anergic clones
			memory potential Aβ T cells		FOXO1/Klf2 (CCR7/S1PR1 migration)		Tolerogenic Aβ T cells
		γδ T cells	Death by neglect	γδ T cells	Activation RANTES and CXCR3	γδ T cells	Naive/low-affinity γδ; anergic clones
			γδ selection (PLZF)				Vδ2^+^ phosphoantigen presentation

## Data Availability

The data that support the findings of this study are available upon an email request from the corresponding author, S.V. Sennikov.
